# Fourier Ptychographic Neural Network Combined with Zernike Aberration Recovery and Wirtinger Flow Optimization

**DOI:** 10.3390/s24051448

**Published:** 2024-02-23

**Authors:** Xiaoli Wang, Zechuan Lin, Yan Wang, Jie Li, Xinbo Wang, Hao Wang

**Affiliations:** Electronics Information Engineering College, Changchun University, Changchun 130022, China; wangxl@ccu.edu.cn (X.W.); 210401103@mails.ccu.edu.cn (Z.L.); lij69@ccu.edu.cn (J.L.); wangxb@ccu.edu.cn (X.W.); blueswang@ccu.edu.cn (H.W.)

**Keywords:** Fourier ptychographic microscopy, aberration correction, pupil recovery, Zernike polynomials, Wirtinger Flow

## Abstract

Fourier ptychographic microscopy, as a computational imaging method, can reconstruct high-resolution images but suffers optical aberration, which affects its imaging quality. For this reason, this paper proposes a network model for simulating the forward imaging process in the Tensorflow framework using samples and coherent transfer functions as the input. The proposed model improves the introduced Wirtinger flow algorithm, retains the central idea, simplifies the calculation process, and optimizes the update through back propagation. In addition, Zernike polynomials are used to accurately estimate aberration. The simulation and experimental results show that this method can effectively improve the accuracy of aberration correction, maintain good correction performance under complex scenes, and reduce the influence of optical aberration on imaging quality.

## 1. Introduction

Fourier ptychographic microscopy (FPM) [1,2] is an emerging imaging technique, which was proposed by Zheng et al. in 2013. Compared to the traditional microscopy imaging mode, this technique combines the ideas of phase recovery [3,4,5], stacked imaging [6], and synthetic aperture [7] by breaking through the limitation of the numerical aperture of the objective lens and improving the image resolution under the premise of ensuring the original size of the field of view. However, optical aberration emerges in the actual application process, which imposes certain limitations on the imaging results.

**Figure 1:**
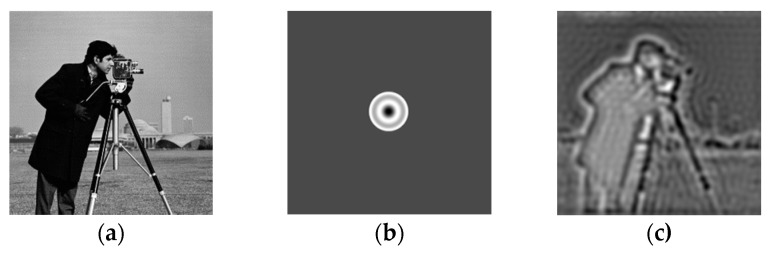
Comparison of images before and after adding aberrations: (**a**) the cameraman image; (**b**) the coherent transfer function with the addition of a spherical aberration; (**c**) the image with the addition of a spherical aberration.

Aberration refers to the difference between the actual and ideal images. While beam focusing in optics can be elaborated as the convergence of light rays to a single point, aberration is the deviation of light rays from the optimal focal point, causing the focus to spread in space [8]. As the imaging system has a certain aperture and field of view, the imaging position for incident light can be different at different apertures. In optics, the aberration in the imaging system can be divided into seven kinds, namely, spherical aberration, coma, dispersion, field curvature, aberration, positional chromatic aberration, and magnification chromatic aberration, as shown in Figure 1.

Aberration is commonly corrected by restoring high-resolution complex objects and unknown aberration pupil functions in the iterative process. For example, Ou et al. [9] proposed a phase recovery algorithm (EPRY-FPM) based on the ePIE method [10], which restores the extended sample spectrum and the pupil function of the imaging system by employing the image of the sample captured by the FPM. With the continuous development of deep learning, more and more researchers have introduced the aberration correction process into the neural network, with the purpose of improving the computational efficiency of the algorithm by taking advantage of its fast computing. For example, using the neural network, Zhang et al. [11] modeled the samples and aberrations as the learnable weights of the multiplication layer and discovered that the INNM network architecture could obtain a complex sample without aberrations. Zhang et al. [12] proposed a Fourier imaging neural network (FINN-CP) with Tensorflow, which is composed of two models, for effectively correcting the position error and wavefront aberration of the system. Hu et al. [13] proposed a microscopic image aberration correction method based on deep learning and aberration prior knowledge, which enhances and corrects the microscopic image in the form of image restoration. Zhang et al. [14] combined the channel attention module with a physics-based neural network to adaptively correct aberrations; Zhao et al. [15] established the relationship between the phase and aberration coefficient through deep learning to segment samples and backgrounds [16] and realized fast automatic aberration compensation correction [17]. Wu et al. [18] proposed an FPM aberration correction reconstruction framework (AA-P) algorithm based on an improved phase retrieval strategy, which improves the iterative reconstruction quality by optimizing the spectral function and the pupil function update strategy while alleviating the influence of mixed wavefront aberrations on the reconstructed image quality and avoiding the occurrence of errors in the reconstruction process. The quality of image reconstruction can be ensured by aberration correction, endowing the reconstructed image with more details. Xiang et al. [19] proposed a phase diversity-based FP (PDFP) scheme for aberration correction. The PD algorithm is an unconventional imaging technique introduced by Gonsalves and Chidlaw [20], which characterize wavefront aberrations by means of a set of focused images and defocused images. Experiments have proven the ability of this scheme to correct changing aberrations and improve image quality. Aberration correction can ensure the quality of image reconstruction, achieving the reconstructed image with more details.

In this paper, we propose an aberration correction method based on the Fourier ptychographic microscopy technique for the aberration existing in the imaging process and name it Integrated Neural Network based on Improved Wirtinger Flow (INN_IWF). The model proposed in this paper is a trainable network constructed on the basis of the TensorFlow framework to simulate the entire process. The network simulates the forward imaging process of the Fourier ptychographic microscopy system while modelling the optical aberration of the objective lens as the optical pupil function to better estimate the optical aberration and optimize the update by back propagation. Furthermore, the alternate updating (AU) mechanism and the Zernike mode are introduced to the model to further improve the performance of the proposed network. Therefore, this method can effectively recover optical aberrations while guaranteeing the overall performance of the network. The results of several sets of experiments show that the mentioned method is superior to other methods in its capability to effectively improve the quality of image reconstruction while retaining more detailed information.

## 2. Methods

### 2.1. Fourier Ptychographic Microscope

**Figure 2:**
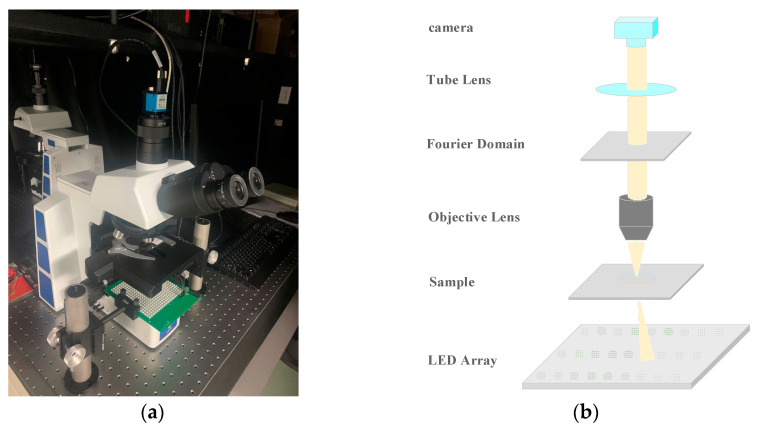
Fourier ptychographic microscope [21]. (**a**) Real FPM system; (**b**) FPM Simulation Schmatic.

The difference between a Fourier ptychographic microscope and conventional microscope is that the Fourier ptychographic microscope uses an array of LEDs instead of a conventional microscope light source. The LEDs are correctly selected to achieve illumination from a variety of angles. Figure 2a shows the RX50 series upright field microscopes and Figure 2b shows the simulation schematic diagram of the device. The camera used in the device is a DMK 33UX264 camera (The Imaging Source, Bremen, Germany, 3.45 µm, 2448 × 2048). The purpose of this device is to digitally image the sample. Optical imaging collected by the device can be accomplished either directly visually or by using the software to view the actual iPlease provide manufacturer and address informationmages captured by the camera.

The device consists of a DMK 33UX264 camera, an eyepiece, an optical path selector lever, a Y-axis moving handwheel, a mirror group, a tightening and loosening adjusting handwheel, an adjusting light wheel, a light collector mirror, an X-axis moving handwheel, a mechanical platform, an LED light board holder, an LED light board (20 × 20), etc. The LED light board parses the commands from the MATLAB program sent through the serial port to light up the LED lights in the specified positions. With LED lights and LED built-in RGB three-color beads, the device can capture images using a black and white camera and synthesize these images into color images. The light panel can be fixed or moved downwards and upwards through LED brackets.

### 2.2. Imaging Model and Reconstruction Model

#### 2.2.1. Imaging Model

In the forward imaging process, the sample can be represented by the transfer function o(r), where *r* represents the two-dimensional coordinate. Assuming that the distance between the LED lamp and the sample is far enough, the illumination wave of the LED lamp can be approximated as an oblique plane wave, and the wave vector of the nth lamp can be expressed as

(1) $$k_{n}=\left(\frac{\sin\theta_{xn}}{\lambda },\frac{\sin\theta_{yn}}{\lambda }\right)(n=1,2,3,\cdot \cdot \cdot \cdot \cdot \cdot ,N_{\mathrm{led}}),$$

where $\left(\theta_{xn},\theta_{yn}\right)$ represents the incident angle of the nth LED lamp, λ is the wavelength of the incident light, and the complex amplitude entering the sample plane is expressed as $e^{ik_{n}r}$. When the nth LED lamp illuminates the sample, the output field after Fourier transform can be expressed as $F\left\{o(r)e^{ik_{n}r}\right\}=O\left(k-k_{n}\right)$. Illuminating the sample using the oblique plane wave with a wave vector is equivalent to the shift $k_{n}$ of the sample spectrum O(k). When passing through the objective lens, the field is lowpass filtered by the pupil function p(k). At this time, the forward imaging process of FPM can be expressed as

(2) $$I_{nc}\left(r\right)={\left|g_{nc}(r)\right|}^{2}={\left|F^{-1}\{p\left(k\right)*(k-k_{n})\}\right|}^{2},$$

where $I_{nc}\left(r\right)$ represents the intensity information on the sensor, $g_{nc}(r)$ represents the complex amplitude distribution on the sensor, $O(k-k_{n})$ represents the sample spectrum illuminated by its plane wave vector $k_{n}$ plane wave, k represents the two-dimensional coordinate, and $F^{-1}$ represents the inverse Fourier transform [22].

#### 2.2.2. Reconstruction Model

In the reconstruction process, FPM obtains a high-resolution complex amplitude distribution $O_{\varepsilon }\left(r\right)=F^{-1}\{O_{\varepsilon }\left(k\right)\}$ by synthesizing images with different frequency domain information. The classical FPM reconstruction algorithm iteratively estimates the complex amplitude image and updates it using the captured intensity image. An iteration can be expressed as

(3) $$g_{n\varepsilon }\left(r\right)=F^{-1}\{p\left(k\right)*O_{\varepsilon }\left(k-k_{n}\right)\},$$

(4) $$p\left(k\right)*O_{\varepsilon }\left(k-k_{n}\right)=F\left\{g_{n\varepsilon }\left(r\right)*\frac{\sqrt{I_{\mathrm{nc}}\left(r\right)}}{\left|g_{n\varepsilon }\left(r\right)\right|}\right\},$$

Equation (3) is used to estimate the high-resolution image relative to each LED light, while Equation (4) updates the high-resolution image by utilizing the captured low-resolution intensity image. The degree of spectral convergence can be known by repeated calculations, and low-resolution images can be used for the initial $g_{n\varepsilon }(r)$. Finally, the estimated spectral $O_{\varepsilon }\{k\}$ is transformed into $O_{\varepsilon }(r)$ by inverse Fourier transform, and the high-resolution image is extracted from $O_{\varepsilon }(r)$.

### 2.3. Integrated Neural Network Based on Improved Wirtinger Flow

#### 2.3.1. Network Architecture

**Figure 3:**
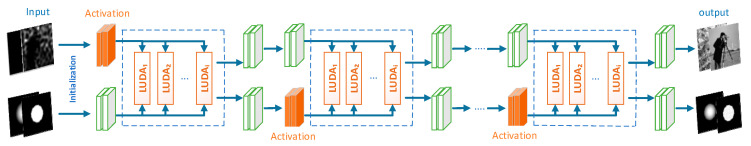
INN_IWF overall flowchart.

**Figure 4:**
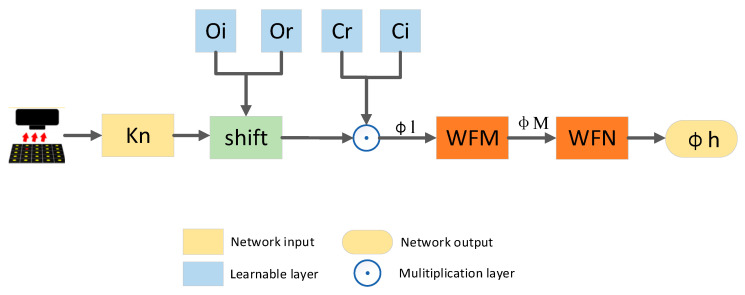
LUDA flowchart.

**Figure 5:**
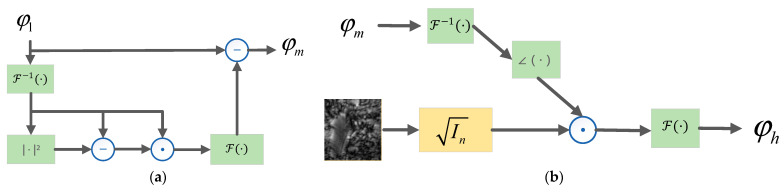
Flowchart of WFM and WFN modules. (**a**) WFM module; (**b**) WFN module.

The whole network implements aberration correction in the Tensorflow framework. Figure 3 shows the overall flowchart of INN_IWF. The sample images captured by up-sampling and the aberration-free coherent transfer function serve as the inputs of the network, respectively. They are alternately updated and fed into the lighting update units with different angles (LUDA), as shown in Figure 4. A set of captured images $I_{n}(r)$ and their corresponding wave vectors $k_{n}(r)$ are taken as a sampling process, and in each sampling, all the samples with different angles are input into the model, and the model parameters are updated by using back-propagation. The expected results are generated through multiple sets of training phases, where the WFM module and the WFN module are separately shown in Figure 5a,b.

The pupil recovery module is specifically formulated as

(5) $$\varphi_{l}\left(k\right)=O\left(k+k_{n}\right)⊙C\left(k\right),$$

(6) $$\varphi_{h}\left(k\right)=F\left\{\sqrt{I_{n}\left(r\right)}⊙f_{WF}(k)\right\},$$

where O(k) is the Fourier function of the sample, and $\varphi_{l}$ and $\varphi_{h}$ are the Fourier original aperture during the update of the INN_IWF network and the updated aperture, respectively. $I_{n}\left(r\right)$ is a pre-upsampled sample image. $C\left(k\right)$ represents the coherence transfer function of the objective lens, which is used to characterize the imaging quality of the diffraction-limited system under the condition of coherent illumination. The standard formula of pupil function CTF can be expressed as

(7) $$C\left(k\right)=\left\{\begin{array}{l} 1,\;\left(k_{x}^{2}+k_{y}^{2}\right)<{(\mathrm{NA}*k_{0})}^{2}\\ \;\;0,\;\;\;\;\;\mathrm{otherwise} \end{array}\right.,$$

where ${(k}_{x},k_{y})$ denotes the two-dimensional spatial coordinates of the Fourier domain, and NA denotes the numerical aperture, $k_{0}=2\pi /\lambda$, where λ is the wavelength of the incident light.

Figure 4 shows the flowchart of LUDA. As the proposed network is defined in the complex domain, the samples and the coherence transfer function (CTF) are divided into real and imaginary parts, which are passed to the network as inputs to LUDA. The samples are shifted according to $k_{n}$ and then multiplied by the CTF to generate $\varphi_{l}(k)$,which is the spectrum before updating, Hence, Equation (5) can be rewritten as

(8) $$\begin{array}{l} \varphi_{l}(k)\;=\left\{O_{r}+j*O_{i}\right\}⊙\left\{C_{r}+j*C_{i}\right\}\\ \;\;\;\;=\left(O_{r}⊙C_{r}-O_{i}⊙C_{i}\right)+j*\left(O_{r}⊙C_{i}-O_{i}⊙C_{r}\right) \end{array}$$

where r and i represent the real and imaginary parts, respectively.

The traditional correction method cannot meet the requirements of complex aberration scenes. Therefore, this paper adds an optimization framework based on the traditional method, as shown in Figure 5a. The whole updating process can be represented by Equation (6). $f_{WF}(k)$ can be expressed by Equation (9).

(9) $$f_{WF}(k)\;=∠\left\{F^{-1}\left\{\varphi_{m}\right\}\right\},$$

where $\varphi_{m}$ is the output of the WFM module in Figure 5a, represented by Equation (10), using the idea of the Wirtinger Flow algorithm [23]. As a technology for solving the phase retrieval problem, the Wirtinger Flow Algorithm [23] will transform the problem into a problem of finding the minimum value and serves as a general optimization framework that can reduce computational costs and effectively deal with noise. The spectrum $\varphi_{l}$ before updating will be divided into two parts, one of which remains unchanged, and the other part is that $\varphi_{l}$ is transformed by inverse Fourier transform and defined as $\varphi_{l}=Y=Ax$, where $A\in C^{m\times n}$ is a linear sampling matrix, which is to be updated through operations such as phase subtraction, the dot product, etc. Then, the updated variable will undergo the Fourier transform again and be subtracted from $\varphi_{l}$ to generate $\varphi_{m}$. The specific flow is shown in the WFM module of Figure 5a.

(10) $${\varphi_{m}}^{\left(k\right)}={\varphi_{l}}^{\left(k\right)}-F\{∆*A^{H}\left[\left({\left|\mathrm{Ax}\right|}^{2}-Y\right)⊙\left(\mathrm{Ax}\right)\right]\},$$

where ∆ is the custom gradient descent step size and ⨀ represents the dot product.

According to Equation (10), $\varphi_{l}\left(k\right)$ is gradient-updated to generate $\varphi_{m}$. $\varphi_{m}$ enters the WFN module for phase conversion for calculating $∠\left\{F^{-1}\left\{\varphi_{m}\right\}\right\}$ to obtain $f_{WF}(k)$. Second, the amplitude of the simulated image in the WFN module is represented by the square root of the pre-sampled intensity image. As an intensity constraint, $f_{WF}(k)$ is multiplied by it. The network generates the updated spectrum $\varphi_{h}\left(k\right)$ according to the update process shown in Figure 5a,b. Since the spectra before and after the update have the same frequency, the whole network structure can be used to obtain the optimal result based on whether the difference between the spectra before and after the update is minimized. In this paper, the mean square error is used to calculate the minimum of the difference between the spectra before and after the update. The loss function is expressed as

(11) $$\begin{array}{l} \mathrm{loss}={\mathrm{loss}}_{\mathrm{MSE}}\\ \;\;=\sum_{i}^{I}{\left(\varphi_{h}\left(k\right)-\varphi_{l}\left(k\right)\right)}^{2}, \end{array}$$

#### 2.3.2. Alternating Update Mechanism

After the above update process, the network outputs the updated samples and CTF. However, the samples and CTF have different properties when the network back propagates, and if the same gradient descent step size is used, the network will fail to converge to a perfect state. Therefore, an alternating update mechanism [24] is adopted to respectively control the gradient descent steps of the samples and CTF in this paper.

The updating process is divided into two parts, one of which aims to change the learning rate of the samples and control the gradient descent step size of the samples while keeping the CTF unchanged, and the other is to change the learning rate of the CTF to control the gradient descent step size of the CTF while keeping the samples unchanged, as identified by orange. Only after these two sections are completed will the network be able to converge to the optimal point and can better results be achieved for the samples and CTF.

#### 2.3.3. Optical Aberration Processing Mechanism

The aberration function of the system is expressed in terms of Zernike polynomials, as shown in Equation (12), which can be used to describe the wavefront characteristics [25].

(12) $$W\left(\rho ,\theta \right)=\sum_{j}a_{j}Z_{j}\left(\rho ,\;\theta \right),$$

where *ρ* and *θ* are variables, $a_{j}$ is the expansion coefficient of different Zernike polynomials, and $Z_{j}\left(\rho ,\theta \right)$ is different Zernike polynomials, which can be expressed as:

(13) $$Z_{\mathrm{odd}\;\mathrm{number}\;j}\left(\rho ,\;\theta \right)=\sqrt{2\left(n+1\right)}R_{n}^{m}(\rho )\cos(m\theta )\;m\neq 0,$$

(14) $$Z_{\mathrm{even}\;\mathrm{number}\;j}\left(\rho ,\;\theta \right)=\sqrt{2\left(n+1\right)}R_{n}^{m}(\rho )\sin\left(m\theta \right)\;m\neq 0,$$

(15) $$Z_{j}\left(\rho ,\theta \right)=\sqrt{\left(n+1\right)}R_{n}^{0}(\rho )\;m=0,$$

where *m* and *n* are positive integers with zeros, and n−m≥0 are even numbers; *n* is the highest order *ρ* of the polynomial; *m* is the azimuth frequency; j is the order of the polynomial and is a function of *n* and *m*; and $R_{n}^{m}(\rho )$ can be expressed as:

(16) $$R_{n}^{m}(\rho )=\sum_{s=0}^{\frac{n-m}{2}}\frac{{\left(-1\right)}^{s}\left(n-s\right)!}{s!\left(\frac{n+m}{2}-s\right)!\left(\frac{n-m}{2}-s\right)!}\rho^{n-2s},$$

The CTF is always updated as a whole. The Zernike polynomials are applied to model the phase of the CTF in this paper, which, therefore, can be expressed as

(17) $$∠C\left(k\right)=\sum_{i=1}^{I}c_{i}*Z_{i}\left(k\right),$$

where *I* in Equation (13) is the number of Zernike polynomials and $c_{i}$ is the coefficient of each Zernike polynomial.

The amplitude of the CTF remains updated as a whole, and the final form of the CTF modelling is expressed as

(18) $$C\left(k\right)=\left|c\left(k\right)\right|⊙\exp\left\{j*∠C\left(k\right)\right\},$$

## 3. Experimental Results

### 3.1. Experimental System Setup

The equipment used for the experiments is shown in Section 3.1. A programmable controlled light source element LED and an illumination wavelength of 532 nm were used and placed 100 mm below the sample to provide illumination. In the sample collection process of the FPM device, the LED array is designed into a 15 × 15 LED rectangular area by programming. The rectangular region can be understood as a two-dimensional coordinate. The LED in the upper left corner of the coordinate starts to light up, and the remaining LED lights up in turn according to the coordinates, forming illumination at different angles. LED lights at different angles illuminate the samples placed on the stage. The FPM system used had a numerical aperture of 0.1 and was used to capture low-resolution sample images illuminated at different angles and record light intensity images using a CMOS camera with a pixel size of 3.45 µm. The results obtained by the INN_IWF were verified through both simulated and real datasets and then compared with those of other methods, such as those proposed by Jiang et al. [26].

Two metrics, namely, the Peak-Signal-to-Noise Ratio (PSNR) and Structural Similarity (SSIM), were used for evaluating the image quality. The Peak-Signal-to-Noise Ratio (PSNR) is an indicator commonly used to measure signal distortion. The larger the PSNR value, the better the image quality. In the field of image evaluation, the Peak-Signal-to-Noise ratio is calculated by the mean squared error (MSE):

(19) PSNR=−10∗logMSE,

The MSE is defined as

(20) $$MSE=mean({\left(I_{1}-I_{2}\right)}^{2}),$$

Among them, $I_{1}$ and $I_{2}$ represent the real image and the contrast image, respectively.

The Structural Similarity Index Measure (SSIM) is used to evaluate the image quality from the perspectives of brightness, contrast, and structure, which is in line with the intuitive effect observed by human vision, whose value falls in the range of 0~1:

(21) $$SSIM=\frac{\left(2\mu_{1}\mu_{2}+C_{1})(2\sigma_{12}+C_{2}\right)}{\left(\mu_{1}^{2}+\mu_{2}^{2}+C_{1})(\sigma_{1}^{2}+\sigma_{2}^{2}+C_{2}\right)}$$

where $\mu_{1}$, $\sigma_{1}$ and $\mu_{2}$, $\sigma_{2}$ represent the mean and standard deviation of the two images, respectively; $\sigma_{12}$ is the covariance of the two; and $C_{1}$ and $C_{2}$ are constant and equal.

### 3.2. Comparative Experiments with Simulated Datasets

**Figure 6:**
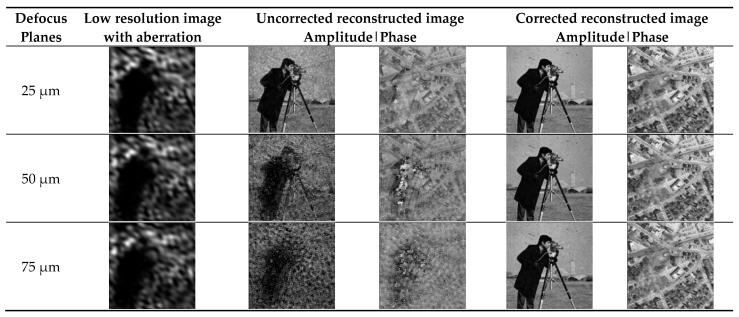
Comparison of low-resolution images with different defocus planes and images before and after aberration correction on sitmulation datasets.

The Cameraman and street map were used as the amplitude and phase images for the simulated dataset, as shown in Figure 6. The optical aberration is dominated by defocus aberration, which is caused by an uneven sample or inaccurate focusing. The experimental equipment, as described above, was used to generate 225 intensity images, from which the amplitude, phase, and CTF were reconstructed.

#### 3.2.1. Correction Performance for Different Defocus Planes

Three defocus planes of 25 µm, 50 µm, and 75 µm were selected to verify the aberration correction performance of the method at different defocus planes (ranging from 25 µm to 75 µm). In this paper, Zernike polynomials were used to estimate the aberration, and the polynomial mode $Z_{2}^{0}$ is about −1.44, corresponding to the defocus aberration of 50 µm. The first column is the low-resolution images with aberrations generated using the forward imaging model, the second and third columns are the images without aberration correction, and the fourth and fifth columns are the images after aberration correction using the INN_IWF network.

Figure 6 demonstrates the effect of aberration on the reconstructed results at different defocus planes. As can be seen from the figure, the effect of aberration on the final generated image became increasingly obvious with the increase in the amount of defocus. Compared with the image without aberration correction, the imaging effect after aberration correction using this method was improved, suggesting that the INN_IWF network can complete the correction of aberrations and maintain a good correction performance on different defocus planes.

**Figure 7:**
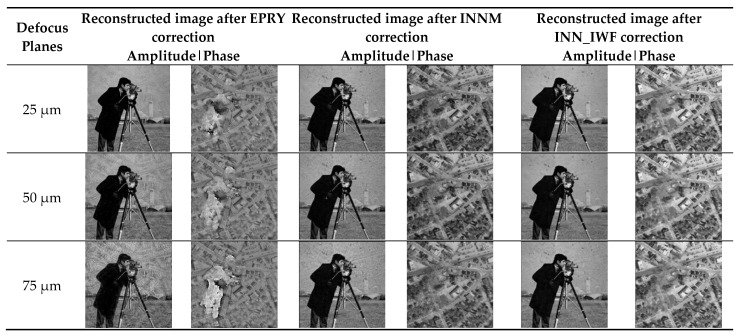
Comparison of the results of different methods on different defocus planes on sitmulation datasets.

In order to further verify the good aberration performance of the proposed method on different defocus planes, INNM [11] and EPRY [9] are used as comparison algorithms in this paper. Several experiments were carried out to compare the correction performance of the above three aberration correction methods on different defocus planes, and the PSNR and SSIM index values calculated by each experiment were averaged. As shown in Figure 7, the images constructed using the three methods were affected to some extent with the increase in the amount of defocus in different defocus planes. Among them, the EPRY method is most affected by the change in the defocus plane, while the method in this paper is least affected by the defocus plane, which can correct the aberration well and obtain the reconstructed image with richer image details. Table 1 is the image reconstruction index values of different methods on different defocus planes, among which the optimal results are marked in bold. In Table 1, the maximum and minimum values of the image reconstruction indexes calculated by many experiments are also shown. The fluctuation range of the maximum and minimum values in Table 1 is smaller than that of the other two methods. The purpose of the maximum and minimum values is to show the fluctuation range of the evaluation indexes of each method. It can be seen from the results shown in Table 1 that the EPRY method has a lower calculated evaluation index value than the other two methods because its correction performance is greatly affected by the change in the defocus plane. The method in this paper adds an optimization process to the network. Compared to the INNM method, it has a better performance and higher image evaluation index value. The above analysis shows that the method put forward in this paper consistently exhibited good aberration correction performances on different defocus planes.

#### 3.2.2. Comparison of the Results of Different Methods on the Simulated Dataset

**Figure 8:**
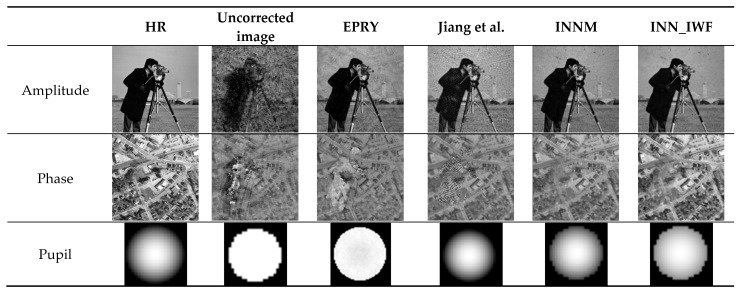
Comparison of the results of different methods on the simulation dataset [26].

The results of this method were compared with those of INNM [11], EPRY [9], and the method proposed by Jiang et al. [26] on a simulated dataset under the condition that defocus aberration was used as the optical aberration, with a size of 50 µm. In addition, the PSNR and SSIM index values for each experimental result of the above methods were calculated and averaged, as shown in Figure 8 and Table 2. The results shown in Figure 8 show that the method in this paper can correct the aberration well. Compared to the other three methods, it has a higher image clarity and more image detail features. In Table 2, the optimal results are marked in bold. Table 2 shows the maximum and minimum values of the image reconstruction indexes calculated by Jiang et al.’s [26] method. The values of other methods are shown in Table 1. The results indicated that the results obtained by the method proposed in this paper were better than those obtained by the other three methods.

### 3.3. Comparative Experiments with a Real Dataset

#### 3.3.1. Correction Performance for Different Defocus Planes

**Figure 9:**
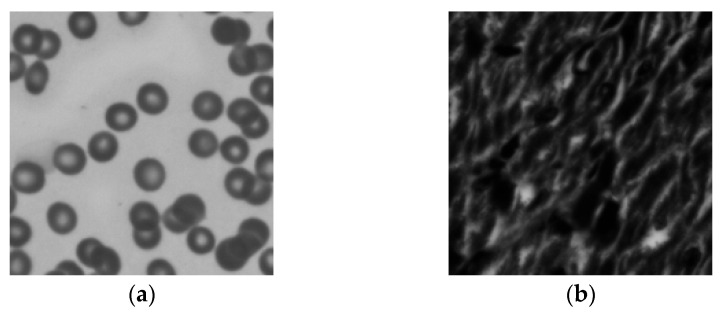
The collected cell images. (**a**) Intensity Image; (**b**) Phase Image.

In order to verify that the proposed method still has a good correction performance in the face of complex aberration conditions, the device of Section 2.1 is used for sample collection. The numerical aperture of the system and the position of the LED array remain unchanged. The 15 × 15 LED illumination array irradiates the real cell image placed on the stage through the plane wave of different angles. The CMOS camera with a pixel size of 3.45 µm captures 225 real sample images with different angles of illumination and records the light intensity image. The intensity and phase images of the real samples are shown in Figure 9a,b.

**Figure 10:**
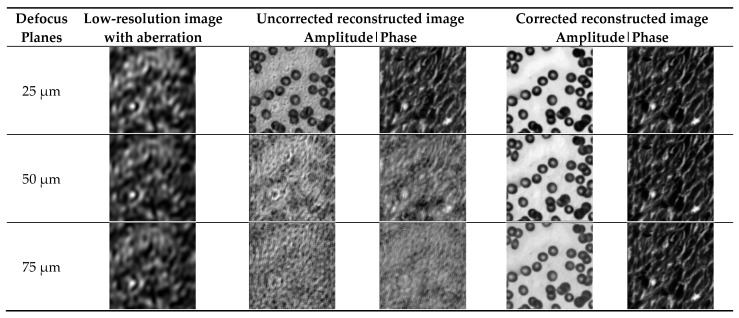
Comparison of low-resolution images with different defocus planes and images before and after aberration correction in a real dataset.

Three defocus planes of 25 µm, 50 µm, and 75 µm were selected for comparison. The aberration correction results for different defocus planes are shown in Figure 10. The first column shows a low-resolution image with aberrations generated using the forward imaging model. The second and third columns are images without aberration correction. The fourth and fifth columns are images after aberration correction using the INN_IWF network.

Figure 10 shows the effect of aberrations on the reconstruction results of cell images at different defocusing planes, which shows that the effect of the aberration on the final reconstructed image became more and more pronounced with the increase in the defocus amount. Compared with the image without aberration correction, the imaging effect of the image corrected by the method proposed in this paper was improved, and the image texture features were retained to a large extent, implying that the INN_IWF network could not only achieve aberration correction but also maintain a good aberration correction performance in the case of severe aberration, so the reconstructed results retained more image detail features.

**Figure 11:**
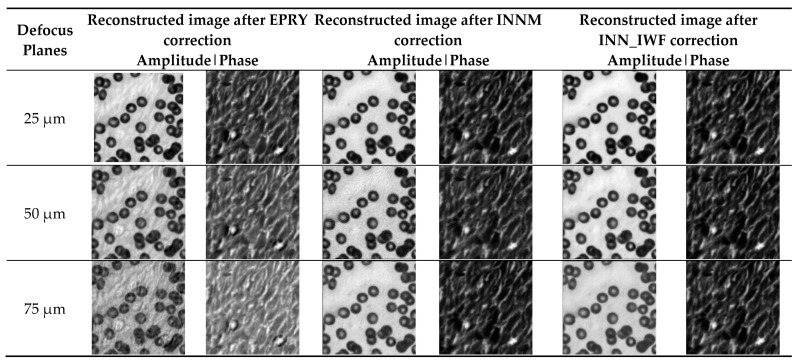
Comparison of the results of different methods on different defocus planes in a real dataset.

In order to further verify the good aberration correction performance of the method presented in this paper for cell images on different defocus planes, INNM [11] and EPRY [9] are used as comparison algorithms. The correction results of the above three aberration correction methods on different defocus planes are compared by multiple experimental results, and the PSNR and SSIM index values calculated by multiple experimental results are averaged. As shown in Figure 11, the defocus amount of different defocus planes gradually increased, which indicates that aberrations on the reconstruction results had a more and more obvious influence on the reconstruction results and that they would also have a certain degree of influence on the reconstruction image quality of the above three methods. The optimal results are in bold in Table 3. The maximum and minimum values of the image reconstruction indexes calculated by multiple experiments are also shown in Table 3. The fluctuation degree of the maximum and minimum values of the proposed method is the same as that of the INNM method, but the numerical value is better than that of the INNM method. As can be seen from the table, aberration had the greatest influence on the EPRY [9] method, and the proposed method and the INNM [11] method are less affected by aberrations. Table 3 also shows that the aberration correction effect of the method proposed on different defocus planes was better than that of the other two methods, with a higher value of the image reconstruction index. The above analysis shows that the proposed method maintains a good aberration correction performance for cell images, and the correction performance is not reduced in complex scenes while retaining image texture features.

#### 3.3.2. Comparison of the Results of Different Methods on a Real Dataset

The dataset used in this subsection is four sets of cell images acquired under real experimental conditions, and the superiority of the method is verified through a comparison with other methods.

**Figure 12:**
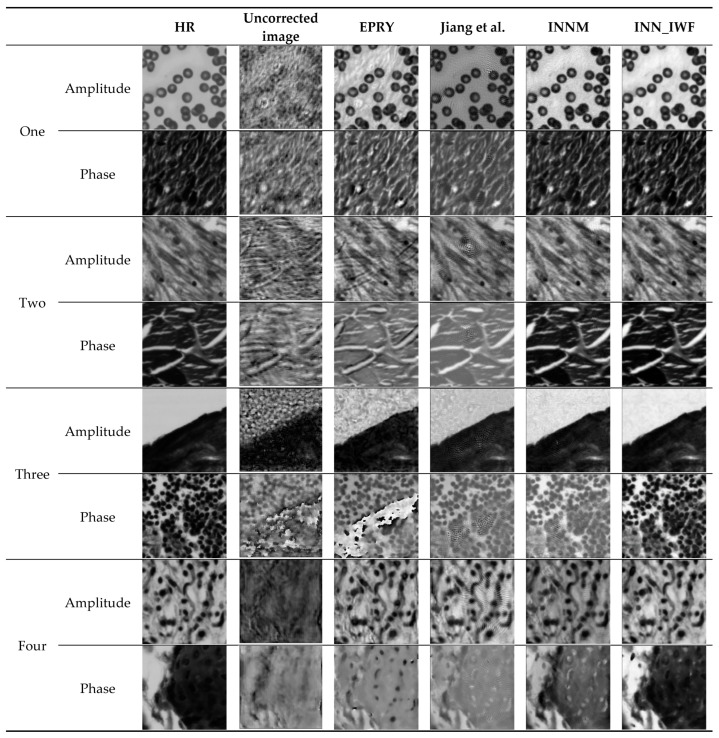
Comparison of the results of different methods in a real dataset [26].

The results of multiple experiments of INN_IWF, INNM [11], EPRY [9], and the method proposed by Jiang et al. [26] in real datasets are compared, as shown in Figure 12. Table 4 is the average value of the image reconstruction index of the above four methods in PSNR and SSIM. Due to the limitation of the table size in Table 4, the maximum and minimum values of multiple sets of real image reconstruction indexes are shown in Table 5. It can be seen from the results that the method in this paper is better than the other methods. The optimal results are in bold. It can be seen from Figure 12 and Table 4 that the image clarity obtained by the INN_IWF was improved when compared with that of the methods proposed by Jiang et al. and EPRY in these four groups of experiments, with more image details. As can been from the reconstruction indexes in Table 4, the reconstruction index value of the method proposed in this paper was higher. The results of the first two groups of experiments were similar to those of the INNM method, while the results of the latter two groups show that the correction performance of the proposed method is better than that of the INNM method. The image reconstruction index values of the two methods in Table 4 show that the INNM is suboptimal. In summary, the method in this paper had a better aberration correction performance in real datasets and was able to obtain better reconstruction results.

## 4. Conclusions

This paper proposes an aberration correction method based on the improved Wirtinger Flow algorithm under the Tensorflow framework. This method simulates the forward imaging process and improves the Wirtinger Flow algorithm introduced into the model, retains the central idea, simplifies the calculation process, and improves the performance of the aberration correction of the network. The alternating update mechanism (AU) updates the sample and the coherent transfer function in batches to obtain better results. Zernike polynomials can estimate aberrations with high precision. The simulation and experimental results show that the INN_IWF network demonstrates a better performance in correcting aberrations while obtaining richer texture details of reconstructed images, proving that the proposed method is superior on different defocus planes, effectively avoiding a low correction accuracy and poor correction performance under complex aberration conditions while retaining more image texture features when compared to traditional algorithms.

## Figures and Tables

**Table 1:** Image reconstruction metrics of different methods on different defocus planes.

	Defocus Planes	EPRY PSNR (dB)/SSIM	EPRY Max/Min Value	INNM PSNR (dB)/SSIM	INNM Max/Min Value	INN_IWF PSNR (dB)/SSIM	INN_IWF Max/Min Value
Amplitude	25 µm	18.04/0.6054	18.76/0.6172 17.30/0.5926	26.40/0.8670	27.5/0.8370 25.3/0.8972	**28.02/0.9214**	**28.29/0.9216** **27.75/0.9182**
	50 µm	17.89/0.5576	18.08/0.5676 17.72/0.5476	22.71/0.8937	22.71/0.8937 21.92/0.8667	**24.97/0.9182**	**24.97/0.9182** **24.26/0.9126**
	75 µm	17.81/0.5003	18.01/0.5546 17.60/0.4462	21.22/0.8622	22.21/0.8698 20.42/0.8544	**23.34/0.9035**	**24.34/0.9035** **22.34/0.9002**
Phase	25 µm	14.51/0.5055	14.99/0.5676 14.02/0.4432	22.02/0.8602	23.02/0.8625 21.04/0.8580	**23.56/0.8776**	**23.56/0.8776** **23.42/0.8726**
	50 µm	14.00/0.4521	14.16/0.4821 13.82/0.4221	21.82/0.8529	21.82/0.8529 20.52/0.8422	**22.73/0.8699**	**22.73/0.8699** **22.36/0.8662**
	75 µm	13.26/0.4024	13.48/0.4455 13.02/0.3592	18.66/0.7928	19.69/0.7921 17.63/0.7935	**21.67/0.8539**	**21.85/0.8639** **21.49/0.8429**

**Table 2:** Image reconstruction metrics of different methods on the simulated dataset.

	EPRY PSNR (dB)/SSIM	Jiang et al. [26] PSNR (dB)/SSIM	Jiang et al. [26] Max/Min Value	INNM PSNR (dB)/SSIM	INN_IWF PSNR (dB)/SSIM
Amplitude	17.89/0.5576	21.88/0.7360	21.97/0.7370 21.79/0.7270	22.71/0.8937	**24.97/0.9182**
Phase	14.00/0.4521	17.01/0.6900	17.07/0.6903 16.94/0.6988	21.82/0.8529	**22.73/0.8699**

**Table 3:** Image reconstruction metrics of different methods on different defocus planes in a real dataset.

	Defocus Planes	EPRY PSNR (dB)/SSIM	EPRY Max/Min Value	INNM PSNR (dB)/SSIM	INNM Max/Min Value	INN_IWF PSNR (dB)/SSIM	INN_IWF Max/Min Value
Amplitude	25 µm	21.76/0.7023	24.76/0.7023 20.76/0.6626	27.29/0.9621	27.60/0.9657 26.98/0.9585	**33.36/0.9838**	**34.36/0.9840** **32.46/0.9752**
	50 µm	20.08/0.6588	21.10/0.6636 20.02/0.6586	22.63/0.9008	23.12/0.9108 22.16/0.8902	**26.23/0.9506**	**27.46/0.9683** **25.96/0.9489**
	75 µm	19.29/0.6182	20.01/0.6282 19.01/0.6084	20.21/0.8254	21.04/0.8355 20.18/0.8153	**21.57/0.8955**	**21.77/0.8977** **21.30/0.8923**
Phase	25 µm	16.52/0.6371	17.42/0.6381 15.62/0.6321	36.14/0.9818	36.43/0.9837 35.85/0.9799	**40.52/0.9916**	**41.15/0.9918** **39.89/0.9901**
	50 µm	12.73/0.5167	13.72/0.5203 11.62/0.4960	29.32/0.9443	29.69/0.9447 28.99/0.9440	**37.64/0.9871**	**37.84/0.9872** **37.44/0.9868**
	75 µm	8.54/0.3893	9.85/0.3996 8.32/0.3792	20.32/0.9309	20.95/0.9329 19.79/0.9288	**34.02/0.9679**	**34.19/0.9743** **33.80/0.9610**

**Table 4:** Image reconstruction metrics of different methods in a real dataset.

		EPRY PSNR (dB)/SSIM	Jiang et al. [26] PSNR (dB)/SSIM	INNM PSNR (dB)/SSIM	INN_IWF PSNR (dB)/SSIM
One	Amplitude	20.08/0.6588	12.34/0.7732	22.63/0.9008	**26.23/0.9506**
	Phase	12.73/0.5167	15.83/0.7298	29.32/0.9443	**37.64/0.9871**
Two	Amplitude	22.15/0.7915	22.20/0.8784	29.89/0.9385	**31.61/0.9690**
	Phase	11.28/0.5481	10.57/0.5301	30.57/0.9297	**33.61/0.9724**
Three	Amplitude	20.01/0.6948	20.56/0.7687	22.11/0.8647	**26.09/0.9335**
	Phase	9.56/0.4883	13.22/0.6115	12.70/0.6383	**25.86/0.9176**
Four	Amplitude	22.70/0.8253	18.85/0.8183	19.98/0.7986	**22.80/0.9246**
	Phase	9.54/0.4568	9.89/0.5940	12.86/0.6086	**21.09/0.8544**

**Table 5:** The maximum and minimum values of the reconstruction metrics of different methods in a real dataset.

		EPRY PSNR (dB)/SSIM Max/Min Value	Jiang et al. [26] PSNR (dB)/SSIM Max/Min Value	INNM PSNR (dB)/SSIM Max/Min Value	INN_IWF PSNR (dB)/SSIM Max/Min Value
One	Amplitude	21.10/0.6636 20.02/0.6586	13.34/0.7743 11.32/0.7682	23.12/0.9108 22.16/0.8902	**27.46/0.9683** **25.96/0.9489**
	Phase	13.72/0.5203 11.62/0.4960	16.12/0.7328 15.54/0.7268	29.69/0.9447 28.99/0.9440	**37.84/0.9872** **37.44/0.9868**
Two	Amplitude	22.15/0.7916 22.10/0.7901	22.26/0.8884 22.12/0.8682	29.99/0.9398 29.69/0.9372	**33.29/0.9752** **29.93/0.9629**
	Phase	11.32/0.5483 11.25/0.5476	11.02/0.5408 10.57/0.5401	31.01/0.9299 30.12/0.9293	**36.61/0.9793** **30.62/0.9655**
Three	Amplitude	20.80/0.6956 19.21/0.6946	20.90/0.7736 20.18/0.7636	22.21/0.8699 22.01/0.8595	**26.29/0.9342** **26.02/0.9330**
	Phase	9.86/0.4896 9.24/0.4824	13.54/0.6215 12.90/0.6015	13.01/0.6434 12.39/0.6332	**26.10/0.9207** **25.53/0.9146**
Four	Amplitude	22.74/0.8262 22.67/0.8221	19.30/0.8283 18.38/0.8083	20.01/0.7987 19.88/0.7985	**22.96/0.9268** **22.72/0.9246**
	Phase	9.60/0.4589 9.42/0.4558	9.90/0.5946 9.86/0.5938	13.06/0.6099 12.67/0.6072	**21.42/0.8568** **21.02/0.8542**

## Data Availability

Data sharing is not applicable to this article.
